# Supplementary material for: Splice-Junction-Based Mapping of Alternative Isoforms in the Human Proteome
Source: Cell Rep. Author manuscript; Available in PMC 2020 Jan 15. (PMC6961840; doi:10.1016/j.celrep.2019.11.026)
Supplement: 2 [file NIHMS1546469-supplement-2.zip › DF1/PXD009737/TestisTrypsin/TestisTrypsin_3_CLTCL1_LQEHFQLQNLGINPANIGFSTLTMESDK.pdf]

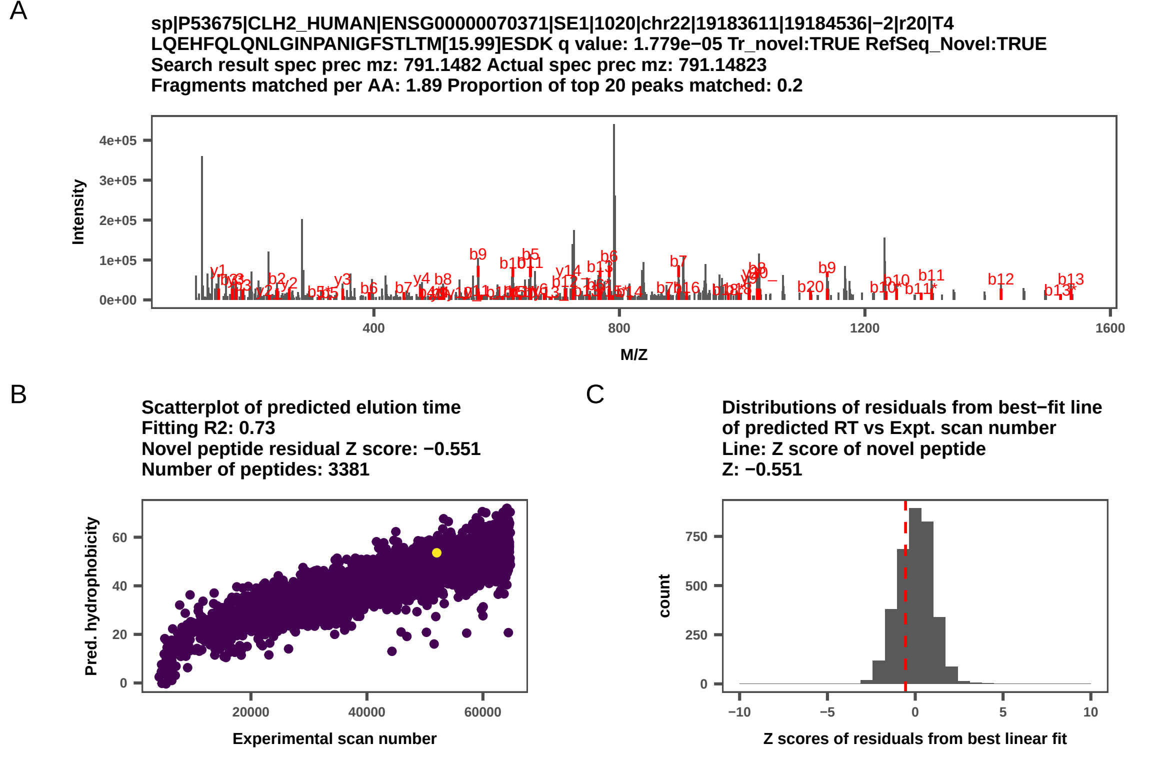
A
sp|P53675|CLH2_HUMAN|ENSG00000070371|SE1|1020|chr22|19183611|19184536|−2|r20|T4
LQEHFQLQNLGINPANIGFSTLTM[15.99]ESDK q value: 1.779e−05 Tr_novel:TRUE RefSeq_Novel:TRUE
Search result spec prec mz: 791.1482 Actual spec prec mz: 791.14823
Fragments matched per AA: 1.89 Proportion of top 20 peaks matched: 0.2
Intensity
4e+05
3e+05
2e+05
1e+05
0e+00
400
800
1200
1600
M/Z
B
Scatterplot of predicted elution time
Fitting R2: 0.73
Novel peptide residual Z score: −0.551
Number of peptides: 3381
Pred. hydrophobicity
60
40
20
0
20000
40000
60000
Experimental scan number
C
Distributions of residuals from best−fit line
of predicted RT vs Expt. scan number
Line: Z score of novel peptide
Z: −0.551
count
750
500
250
0
−10
−5
0
5
10
Z scores of residuals from best linear fit
